# Hiding in the dark: pan-cancer characterization of expression and clinical relevance of CD40 to immune checkpoint blockade therapy

**DOI:** 10.1186/s12943-021-01442-3

**Published:** 2021-11-10

**Authors:** Chi Yan, Ann Richmond

**Affiliations:** 1 Tennessee Valley Healthcare System, Department of Veterans Affairs, Nashville, TN USA; 2 Department of Pharmacology, Vanderbilt University School of Medicine, 432 PRB, 2220 Pierce Ave, Nashville, TN 37232 USA

**Keywords:** CD40, Cancer, Nevi, Melanoma, PD-1/PD-L1, CTLA4, Targeted therapy, Immune therapy

## Abstract

- *CD40* expression correlates with the type I anti-tumor response and better survival.
- Pan-cancer bioinformatics characterization reveals reduced *CD40* expression in 11 cancer types, including *RAS*^*mut*^ melanoma compared to nevi.
- *RAS* mutation correlates with reduced *CD40* expression in malignant melanoma.
- *CD40* expression is associated with better response to immune checkpoint blockade therapy in melanoma.

**Supplementary Information:**

The online version contains supplementary material available at 10.1186/s12943-021-01442-3.

## Main text

CD40, also known as TNFRSF5, is a co-stimulatory cell surface receptor present on antigen-presenting cells (APC), non-hematopoietic cells (e.g., myofibroblasts, fibroblasts, epithelial, and endothelial cells) and tumor cells [1, 2]. The CD40 and CD40 ligand (CD40LG) interaction mediates anti-tumoral immune responses by increasing immunogenic cell death (ICD) of tumor cells, APC activation, tumor immunogenicity by up-regulating major histocompatibility complex (MHC) molecules, production of proinflammatory factors, costimulation of CD4^+^ and CD8^+^ T cells, and tumor cell susceptibility to T-cell lysis [2–4]. Recently, our group demonstrated that RAS/RAF/PI3K pathway inhibition promotes ICD and augments response to immune checkpoint blockade (ICB), which mechanistically relies on CD40 induction by melanoma cells [2]. While the prevalence and importance of RAS/RAF/PI3K activating mutations across a very broad spectrum of human cancer types is clear, we still lack understanding of whether CD40 regulation by the RAS/RAF/PI3K pathway broadly serves as an immune evasion/tolerance mechanism in tumor progression and response to therapy. In this study, we systematically characterized the expression and clinical relevance of CD40 across a variety of normal tissues and cancer types.

## CD40 expression correlates with the type-I anti-tumor response and better clinical outcome

**Fig. 1:**
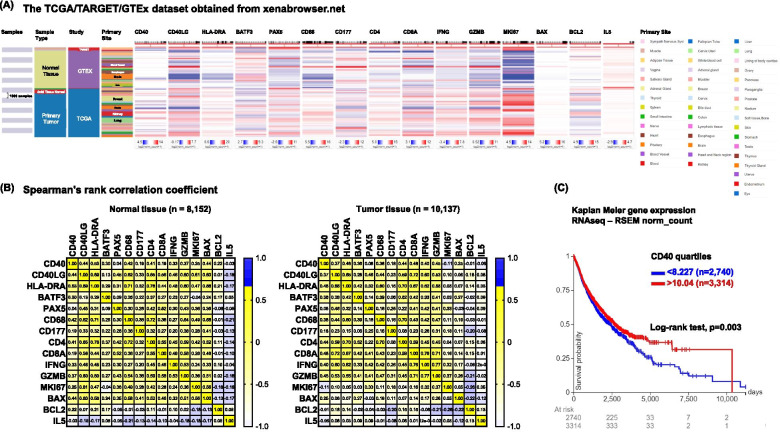
CD40 expression is positively correlated with type I anti-tumor T-cell responses and better survival. **A** Heat map of indicated gene expression across normal and tumor tissues in the TCGA/TARGET/GTEx database obtained from the UCSC Xena web-based tool. **B** Correlation matrix with Spearman’s rank correlation coefficients of RNA-Seq normalized count of indicated genes. **C** Kaplan Meier survival plot with log-rank test of pan-cancer samples stratified by CD40 quartiles of RNA-Seq normalized count

Using the TCGA/TARGET/GTEx RNA-Seq dataset which covers 23 normal tissues and corresponding cancer types (Fig. 1A), we analyzed *CD40* mRNA expression and its association to its ligand (*CD40LG)*, markers of MHC (*HLA-DRA*), dendritic cells (*BATF3*), B cells (*PAX5*), macrophages (*CD68*), granulocytes (mainly neutrophils, *CD177*), T-cells (*CD4* and *CD8A*), type-I anti-tumor responses (*IFNG* and *GZMB*), cell proliferation (*MKI67*), cell death (*BAX*), cell survival (*BCL2*) and type-II pro-tumor responses (*IL5*) in normal (*n* = 8152) and cancer (*n* = 10,137) tissues (Fig. 1B). Using Spearmen *r* ≥ 0.3 or *r* ≤ − 0.3 and *p* < 0.001 as a cut-off, we showed that *CD40* is moderately co-expressed with *CD40LG* in the normal tissues (*r* = 0.44) and in the tumor microenvironment (TME, *r* = 0.37). In normal tissues, *CD40* is positively associated with type-I responses (*IFNG*, *r* = 0.33 and *GZMB*, *r* = 0.37) and response to antigen-presentation (*HLA-DRA*, *r* = 0.53), T helper (Th) cells (*CD4*, *r* = 0.41), but not cytotoxic T cells (*CD8A*, *r* = 0.19). Additionally, the closest association of cell proliferation (*MKI67*) is with *CD40LG* (*r* = 0.61) and pro-apoptotic marker *BAX* (*r* = 0.56) [5], suggesting that the CD40/CD40LG axis is important in the process of immune cell turnover and homeostasis under normal conditions. We found that *CD68* (macrophage), but not *CD177* (neutrophil), is positively correlated with *CD40* in both normal (*r* = 0.42) and tumor (*r* = 0.38) tissues. In addition, *CD68* (macrophage) is highly correlated to antigen-presentation responses (*HLA-DRA*, *r* = 0.71 and 0.60 in normal and tumor tissues, respectively) and moderately correlated to *GZMB* (*r* = 0.54 and 0.47 in normal and tumor tissues, respectively). In the TME, we found that *CD40* expression is moderately correlated with type-I T-cell and APC responses as reflected by significant coexpression with *HLA-DRA* (*r* = 0.46), *CD4* (*r* = 0.44), *CD8A* (*r* = 0.48), *IFNG* (*r* = 0.40) and *GZMB* (*r* = 0.45). Furthermore, *CD40* is moderately correlated with *BATF3* (dendritic cells) in normal (*r* = 0.30) and tumor (*r* = 0.36) tissues. In contrast, *CD40LG* is moderately correlated with *PAX5* (B cells, *r* = 0.45 and 0.46 in normal and tumor tissues, respectively), but not *BATF3* (dendritic cells). In tumors, pan-APC marker *HLA-DRA* (MHC molecule), *BATF3* (dendritic cells), *PAX5* (B cells) and *CD68* (macrophages) exhibited a positive correlation (*r* > 0.3) with *CD8A*, *IFNG* and *GZMB*. Together, these results suggested that while the CD40/CD40LG signal may be provided by APCs in a context-dependent manner, it is associated with beneficial T-cell-mediated cytotoxic effects (*CD8A*, *IFNG* and *GZMB*). The observed association between *CD40* expression and the type-I anti-tumor T-cell response could partially result from CD40^+^CD8^+^ T cells in the TME, since CD40 activation is fundamental for their memory generation [6]. There is no correlation between *CD40/C40LG* and the markers of cell death (*BAX*) or cell survival (*BCL2*) in the TME. In addition, the CD40/CD40LG axis exhibited no correlation with type-II pro-tumor responses (*IL5*) in either normal or cancer tissues. Consistently, high *CD40* expression in tumors is associated with a better survival of cancer patients (Fig. 1C, log-rank test, *p* = 0.003).

## CD40 regulation and connection to RAS/RAF/PI3K-targeted and immune therapies

**Fig. 2:**
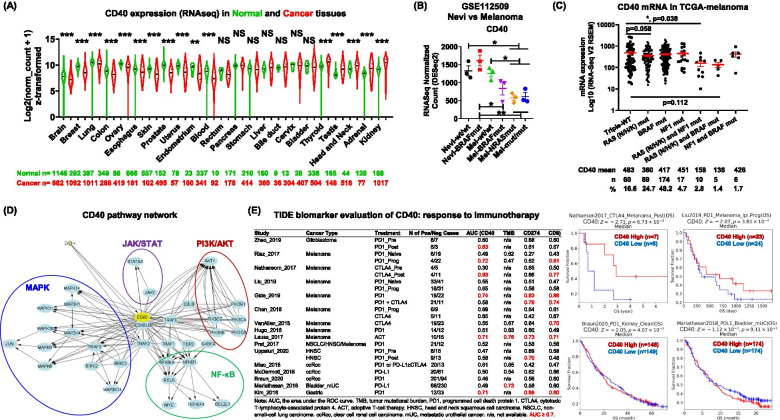
Pan-cancer characterization of CD40 expression and pathway network and its predictive value in therapeutic response to immune checkpoint blockade. **A** CD40 expression in human normal and tumor tissues statistically evaluated by the Mann-Whitney test. **B** CD40 expression in human nevi and melanoma samples in the GSE112509 dataset. **C** CD40 expression in Skin Cutaneous Melanoma (TCGA, Firehose Legacy, *n* = 479) dataset. **D** Human CD40 pathway network derived from the Pathway Interaction Database which is curated by NCI/Nature. **E** TIDE biomarker evaluation and Kaplan Meier survival plots of CD40 in response to immunotherapy in cancer

After identifying the anti-tumoral role of CD40/CD40LG in pan-cancer analysis, we next explored whether any cancer type(s) may alter CD40 in tumorigenesis (Fig. 2A). Among the 23 cancer types screened, *CD40* downregulation was observed in 11 malignancies compared to corresponding normal tissues, including brain, breast, lung, colon, ovary, esophagus, skin, prostate, uterus, endometrium, and blood cancers. In contrast, five malignancies (thyroid, testis, head and neck, adrenal, and kidney) exhibited an increased *CD40* expression in the established tumors. Given that melanocytes comprise a minor population (< 10%) in normal skin [7], the direct comparison between normal skin and melanoma tissues may be biased by the predominant keratinocyte composition in the epidermal basal layer. Thus, we further validated the *CD40* expression in nevus samples and compared it to expression in melanoma tissues using the NCBI-GSE112509 bulk RNA-Seq dataset (Fig. 2B) [8]. There is no significant alteration of *CD40* expression among *NRAS*^*wt*^*/BRAF*^*wt*^ nevi, *NRAS*^*wt*^*/BRAF*^*mut*^ nevi and *NRAS*^*wt*^*/BRAF*^*wt*^ melanoma tissues. However, we observed heterogeneity of *CD40* expression among the melanoma tissues bearing oncogenic *NRAS* and *BRAF* mutations. A reduced *CD40* expression was observed in *NRAS*^*wt*^*/BRAF*^*mut*^ melanoma compared to *NRAS*^*wt*^*/BRAF*^*mut*^ nevus tissues. Furthermore, *NRAS*^*mut*^ melanoma exhibited a reduced *CD40* expression compared to either *NRAS*^*wt*^*/BRAF*^*wt*^ nevi, *NRAS*^*wt*^*/BRAF*^*mut*^ nevi or *NRAS*^*wt*^*/BRAF*^*wt*^ melanoma tissues. Consistently, *RAS (N/H/K)* mutation alone (*p* = 0.058), or in combination with *NF1* mutation (*p* = 0.038), was associated with a reduced *CD40* expression in TCGA-melanoma (Firehose Legacy database, *n* = 479) (Fig. 2C). These results match well with the observation that RAS/RAF/PI3K pathway inhibition increased CD40 expression in melanoma cells [2].

We next explored the pathway network of human CD40 based on the Pathway Interaction Database curated by NCI/Nature (http://www.ndexbio.org/). The CD40/CD40LG axis may lead to the activation of MAPK, PI3K/AKT, NF-κB and JAK3/STAT5 pathways (Fig. 2D, clustering coefficient = 0.379), which are known RAS-downstream signaling effector pathways [9]. Thus, when therapies that inhibit the RAS/RAF/PI3K pathway are utilized, CD40 can be upregulated, allowing CD40-mediated compensatory activation of the RAS-downstream signaling activity. Also, human melanoma cells with increased CD40 at either copy number or mRNA expression levels are significantly more sensitive to the RAF inhibitor treatments [2]. The compensatory regulation of CD40 signaling mediated by the RAS/RAF/PI3K-pathway might be utilized in tumors with low intrinsic CD40 levels, such as *NRAS*^*mut*^ melanoma. Indeed, MEK inhibition (MEKi) increased survival of *NRAS*^*mut*^ melanoma patients [10]. The results from the NEMO trial comparing the efficacy of a MEK inhibitor (MEK162) versus dacarbazine in unresectable or metastatic *NRAS*^*mut*^ melanoma revealed that the MEK162 treatment group had 2.8-month PFS compared to the chemotherapy group with a 1.5-month survival. However, when the analysis was confined to patients who had received prior immunotherapy, the MEK162 PFS was 5.5 months compared to 1.6 months for the chemotherapy treatment group. These data further support the notion that *NRAS*^*mut*^ melanoma patients who received first-line immune checkpoint blockade therapy might benefit from MEKi therapy [11, 12].

Recent advances in ICB therapy, such as targeting programmed cell death protein 1 (PD-1)/PD-ligand 1 (PD-L1) and/or cytotoxic T-lymphocyte-associated protein 4 (CTLA-4), illustrate powerful enhancement of the patient’s anti-tumor immune responses [13]. Currently, ICB is a first-line therapy for several cancer types, including unresectable metastatic melanoma. To evaluate whether there might be a predictive value of CD40 for immunotherapy, we utilized the Tumor Immune Dysfunction and Exclusion (TIDE) framework (tide.dfci.harvard.edu), which is a computational framework developed to evaluate potential of tumor immune escape based on the gene expression profiles of cancer samples. The TIDE framework data base covers glioblastoma (*n* = 24), head and neck squamous cell carcinoma (HNSC, *n* > 45), non-small-cell lung carcinoma (NSCLC, *n* < 33), kidney cancer (*n* = 408), bladder cancer (*n* = 298), gastric cancer (*n* = 45) and 12 melanoma cohorts (*n* > 426). We analyzed the area under the ROC curve (AUC) for CD40 expression in comparison with existing biomarker signatures, including tumor mutational burden (TMB), CD274 (PD-L1) and CD8 [14], as a tool for predicting response to immunotherapy (Fig. 2E). The prediction performances of CD40 are comparable to CD8 in ≥50% melanoma cohorts and one gastric cancer cohort, where both biomarkers exhibited an AUC > 0.7, predicting a strong likelihood of positive response to immunotherapy. Notably, CD40 is the only biomarker that achieved a good performance in predicting ICB response in glioblastoma (AUC = 0.83). TMB (AUC = 0.73) and CD274 (AUC = 0.7) are predictive for ICB response in one bladder cancer cohort and one HNSC cohort, respectively. None of these biomarkers exhibited good performance in NSCLC or kidney cancer. All the biomarkers in the pre-treatment or treatment-naive biopsies from the cancers evaluated here (Fig. 2E) had a prediction score lower than 0.7, suggesting that these biomarkers may be more predictive of treatment-induced responses. In general, the predictive ability of these transcriptomic biomarkers to immunotherapy response is still currently limited. Notably, high CD40 is associated with better overall survival (OS) in two melanoma cohorts of patients treated with α-CTLA4 (*n* = 15, *p* = 0.0067) or α-PD1 (*n* = 47, *p* = 0.0381). Nevertheless, there is either no correlation or a negative correlation of CD40 expression level and OS of patients who received α-PD-L1 in bladder cancer (*n* = 348, *p* = 0.9110) or patients who received α-PD1 in kidney cancer (*n* = 295, *p* = 0.0407). The cancer-type-specific predictive performance of CD40 in ICB response suggests additional features of the TME may be involved in bladder and kidney cancers.

There are limitations to our study. For instance, the analysis of mRNA markers and immune profiling in tumors are derived from selected databases and should be interpreted with caution; future functional characterization and validation on the role of CD40 in immune cells versus non-immune cells will be important to determine whether CD40 is a driver or bystander in the specific experimental settings [15]. In the survival analysis using TIDE biomarker evaluation on CD40, results were dichotomized at cohort-specific median of z-scores, which warrants future trials in a larger cohort to validate the role of CD40 in treatment responses to patient’s survival and additional clinical characteristics, e.g., metastatic status. In general, the effect of CD40 on tumorigenesis and response to therapy (sensitivity and toxicity) may largely depend on the context of the tumor type and cancer stage, cytokine availability, ligand/receptor distribution, and crosstalk among different stimuli, cell types, and signaling pathways.

## Conclusion

We have shown a close correlation of the CD40/CD40LG axis with type-I anti-tumor T-cell responses in cancer. The prevalence of reduced CD40 expression in tumor versus normal tissue was identified in many cancer types, including melanoma. *RAS (N/H/K)* mutation correlates with low CD40 expression in melanoma. Moreover, CD40 is a potential predictive biomarker of response to ICB therapy for some tumor types (melanoma, gastric cancer, and glioblastoma).

## Supplementary Information

**Additional file 1.** Supplementary Methods.

## Data Availability

Supplementary Methods can be accessed online.

## Abbreviations

APC: Antigen presenting cells
CD40LG: CD40 ligand
ICD: Immunogenic cell death
MHC: Major histocompatibility complex
ICB: Immune checkpoint blockade
TME: Tumor microenvironment
Th cells: T helper cells
PD-1: Programmed cell death protein 1
PD-L1: PD-1 ligand 1
CTLA-4: Cytotoxic T-lymphocyte-associated protein 4
TIDE: Tumor immune dysfunction and exclusion
HNSC: Head and neck squamous cell carcinoma
NSCLC: Non-small-cell lung carcinoma
AUC: Area under the ROC curve
TMB: Tumor mutational burden
OS: Overall survival
TCGA: The cancer genome atlas
