# Supplementary material for: Hiding in the dark: pan-cancer characterization of expression and clinical relevance of CD40 to immune checkpoint blockade therapy
Source: Mol Cancer. 2021 Nov 10;20:146. doi: 10.1186/s12943-021-01442-3 (PMC8582157; doi:10.1186/s12943-021-01442-3)
Supplement: Supplementary file 1 — Additional file 1. Supplementary Methods. [file 12943_2021_1442_MOESM1_ESM.docx]

**Supplementary Methods**

***UCSC and cBioportal cancer genomics browser analysis***

Functional genomic data sets for correlations between genomic and phenotypic variables were accessed on 21 June 2021 and analyzed from the TCGA (The Cancer Genome Atlas Program) /TARGET (Tumor Alterations Relevant for GEnomics-driven Therapy) /GTEx (Genotype-Tissue Expression) project on the USCS Xena Sever (https://xena.ucsc.edu/) [1]. Data from the TCGA/TAGET/GTEx project is from the UCSC RNA-seq Compendium, where TCGA, TARGET, and GTEx samples are re-aligned and re-analyzed by the same RNA-seq pipeline using RSEM and Kallisto methods. Since all samples are processed using a uniform bioinformatic pipeline, batch effect due to different computational processing is eliminated. Tumor data covering >50 cancer types are obtained from TCGA and TARGET databases from Xena. Two sources of normal expression data were obtained in Xena. The first is normal samples from TCGA, called "solid tissue normal", which are adjacent normal tissues near the tumor. The second is from GTEx samples, called “normal tissue”, which have expression data from normal tissue of individuals who do not have cancer. We identified 23 cancer types with available transcriptome data and corresponding normal tissue data (n>5 per group). The coordinated TCGA/TAGET/GTEx samples (normal tissue, n=8,152; cancer tissue, n=10,137) were utilized for the gene expression analysis and samples of “cell line”, “metastatic” and “samples with null” were excluded. The corresponding Kaplan-Meier plot of overall survival stratified by the quartiles of normalized *CD40* counts were constructed by Xena platform. GraphPad’s Prism 8.0.1 software was used to construct the gene expression matrix of Spearman correlation. To validate *CD40* mRNA expression in human melanoma, genetic mutation status of *RAS (N/H/K)*, *BRAF* and *NF1* was retrieved on 8 September 2021 from Skin Cutaneous Melanoma (TCGA, Firehose Legacy) dataset in cbioportal.org (471 patients / 479 samples) [2].

***RNA-Seq analysis of CD40 in nevi and melanoma samples***

The original data are available at NCBI Gene Expression Omnibus (GEO) under accession number GSE112509. The raw RNA-Seq reads have been extracted from the Sequence Read Archive (SRA) files and converted into FASTQ files in Galaxy version 21.05.1.dev0 [3]. Nevi and melanoma samples were grouped by the genetic mutation status at *NRAS* and *BRAF* genes. The melanoma on nevi samples were excluded from the study. There are three NRAS^wt^BRAF^wt^ nevi samples available in the dataset, and therefore, three samples in each group were randomly selected and retrieved for the subsequence analysis. NRAS^wt^BRAF^wt^ nevi samples: SRR6916932, SRR6916933 and SRR6916934. NRAS^wt^BRAF^mut^ nevi samples: SRR6916886, SRR6916894 and SRR6916926. NRAS^wt^BRAF^wt^ melanoma samples: SRR6916937, SRR6916957 and SRR6916958. NRAS^mut^BRAF^wt^ melanoma samples: SRR6916903, SRR6916917 and SRR6916964. NRAS^wt^BRAF^mut^ melanoma samples: SRR6916918, SRR6916920 and SRR6916946. NRAS^mut^BRAF^mut^ melanoma samples: SRR6916902, SRR6916905 and SRR6916923. Single-end FASTQ files were aligned to hg38 using HISAT2 [4]. Aligned reads were counted using htseq-count with the GENCODE human .gtf file release 38 (GRCh38.p13) as a reference [5]. Differential mRNA gene expression was determined and normalized with the DESeq2 tool [6] using default parameters and annotated in reference to the GENCODE human .gtf file release 38 (GRCh38.p13).

***Human CD40 pathway network***

The human CD40 pathway is derived from the latest BioPAX3 version of the Pathway Interaction Database (PID) curated by NCI/Nature. The PID is a highly-structured, curated collection of information about known bio-molecular interactions and key cellular processes assembled into signaling pathways. The CD40-CD40L signaling dataset (Nodes: 35; Edges: 150) is publicly available from via the NDEx database (<http://www.ndexbio.org/>) [7], which was obtained on 18 June 2021. The original OWL file was first converted to Extended Binary SIF (EBS) by the PAXTools v5 utility. It was then processed to remove redundant edges, node display names were set to official gene symbols and the new NDEx Default Visual Style was finally applied. Cytoscape version 3.8.2 was used to visualize and analyze the pathway network [8]. Average number of neighbors = 7,771; Clustering coefficient = 0.379; Network density = 0.114.

***TIDE biomarker evaluation analysis***

Tumor Immune Dysfunction and Exclusion (TIDE, http://tide.dfci.harvard.edu/) was used to estimate immunotherapeutic response prediction on June 17^th^, 2021 [9]. Twenty-three cohorts with available data on response to immune checkpoint blockade therapy were identified and included for the investigation. The area under the ROC curve (AUC) for gene signatures were generated to indicate the prediction performance of CD40 in comparation with existed biomarkers, including tumor mutational burden (TMB), CD274 (PD-L1) and CD8 [10]. The performance of a random predictor (AUC = 0.5) is represented by the dashed line. The corresponding Kaplan-Meier plots of overall survival were constructed by TIDE platform in patients with melanoma. The P value was calculated by testing the association between prediction scores and overall survival with the two-sided Wald test in a Cox-PH regression [11].

***Statistical analysis***

Effects in standard two-group experiments were compared using a two-sample Mann-Whitney U test between independent samples. A one-way analysis of variance (ANOVA) with post hoc Tukey's HSD test was used for more than two group experiments to compare CD40 gene expression differences. Survival curves are estimated using the Kaplan-Meier method and compared statistically using the log-rank test. Spearman’s rank correlation was used to measure the strength and direction of a monotonic association between two ranked variables. All tests of statistical significance were two-sided. If not otherwise stated, GraphPad’s Prism 8.0.1 software was used for the statistical analysis. Data are presented with median with quartiles. *p ≤ 0.05; **p ≤ 0.01; ***p ≤ 0.001; NS, not significant.

***References for Supplementary Methods***

1. Goldman MJ, Craft B, Hastie M, Repecka K, McDade F, Kamath A, Banerjee A, Luo Y, Rogers D, Brooks AN, et al: **Visualizing and interpreting cancer genomics data via the Xena platform.** *Nat Biotechnol* 2020, **38:**675-678.

2. Cancer Genome Atlas N: **Genomic classification of cutaneous melanoma.** *Cell* 2015, **161:**1681-1696.

3. Goecks J, Nekrutenko A, Taylor J, Galaxy T: **Galaxy: a comprehensive approach for supporting accessible, reproducible, and transparent computational research in the life sciences.** *Genome Biol* 2010, **11:**R86.

4. Kim D, Langmead B, Salzberg SL: **HISAT: a fast spliced aligner with low memory requirements.** *Nat Methods* 2015, **12:**357-360.

5. Anders S, Pyl PT, Huber W: **HTSeq--a Python framework to work with high-throughput sequencing data.** *Bioinformatics* 2015, **31:**166-169.

6. Love MI, Huber W, Anders S: **Moderated estimation of fold change and dispersion for RNA-seq data with DESeq2.** *Genome Biol* 2014, **15:**550.

7. Pratt D, Chen J, Pillich R, Rynkov V, Gary A, Demchak B, Ideker T: **NDEx 2.0: a clearinghouse for research on cancer pathways.** *Cancer Res* 2017, **77:**e58-e61.

8. Shannon P, Markiel A, Ozier O, Baliga NS, Wang JT, Ramage D, Amin N, Schwikowski B, Ideker T: **Cytoscape: a software environment for integrated models of biomolecular interaction networks.** *Genome Res* 2003, **13:**2498-2504.

9. Fu J, Li K, Zhang W, Wan C, Zhang J, Jiang P, Liu XS: **Large-scale public data reuse to model immunotherapy response and resistance.** *Genome Med* 2020, **12:**21.

10. Litchfield K, Reading JL, Puttick C, Thakkar K, Abbosh C, Bentham R, Watkins TBK, Rosenthal R, Biswas D, Rowan A, et al: **Meta-analysis of tumor- and T cell-intrinsic mechanisms of sensitization to checkpoint inhibition.** *Cell* 2021, **184:**596-614 e514.

11. Jiang P, Gu S, Pan D, Fu J, Sahu A, Hu X, Li Z, Traugh N, Bu X, Li B, et al: **Signatures of T cell dysfunction and exclusion predict cancer immunotherapy response.** *Nat Med* 2018, **24:**1550-1558.
